# Antioxidant Activity of *Stryphnodendron rotundifolium* Mart. Stem Bark Fraction in an Iron Overload Model

**DOI:** 10.3390/foods10112683

**Published:** 2021-11-03

**Authors:** Gerson Javier Torres Salazar, Francisco Junio Dias, Paulo Riceli Vasconcelos Ribeiro, Edy Sousa de Brito, Kirley Marques Canuto, Henrique Douglas Melo Coutinho, Jaime Ribeiro-Filho, Monica Gallo, Domenico Montesano, Daniele Naviglio, Gokhan Zengin, José Galberto Martins da Costa

**Affiliations:** 1 Postgraduate Program in Ethnobiology and Nature Conservation, Regional University of Cariri, Coronel Antônio Luíz Street, 1161-Pimenta, Crato 63105-010, Brazil; timotygertor@yahoo.com (G.J.T.S.); junordias195@gmail.com (F.J.D.); galberto.martins@gmail.com (J.G.M.d.C.); 2 Multi-User Laboratory of Natural Products Chemistry, Embrapa Tropical Agroindustry, Sara Mesquita, no 2.270, Neighborhood Planalto do Pici, Fortaleza 60511-110, Brazil; pauloriceli85@gmail.com (P.R.V.R.); edy.brito@embrapa.br (E.S.d.B.); kirley.canuto@embrapa.br (K.M.C.); 3 Postgraduate Program in Biological Chemistry, Regional University of Cariri, Coronel Antônio Luíz Street, 1161-Pimenta, Crato 63105-010, Brazil; hdmcoutinho@gmail.com; 4 Laboratory of Investigation in Genetics and Translational Hematology, Gonçalo Moniz Institute (IGM), Oswaldo Cruz Foundation (FIOCRUZ), Waldemar Falcão Street, 121, Candeal, Salvador 40296-710, Brazil; jaimeribeirofilho@gmail.com; 5 Department of Molecular Medicine and Medical Biotechnology, University of Naples Federico II, Via Pansini 5, 80131 Naples, Italy; 6 Department of Pharmacy, University of Naples Federico II, Via D. Montesano 49, 80131 Naples, Italy; domenico.montesano@unina.it; 7 Department of Chemical Sciences, University of Naples Federico II, Via Cintia, 4, 80126 Naples, Italy; 8 Department of Biology, Science Faculty, Selcuk University, Campus, 42130 Konya, Turkey; gokhanzengin@selcuk.edu.tr

**Keywords:** antioxidant activity, free radicals, iron overload, *Stryphnodendron rotundifolium*, tannins, *UPLC–ESI-qTOF-MS/MS*

## Abstract

*Stryphnodendron rotundifolium* Mart., popularly known as “barbatimão”, is a plant species traditionally used by topical and oral routes for the treatment of infectious and inflammatory diseases. Considering the well-described antioxidant properties of this species, this study investigated the protective effects of its keto-aqueous extract using an in vitro model of iron overload. Phenolic compounds were quantified and identified by Ultra-Performance Liquid Chromatography coupled with quadrupole Time-Of-Flight Electrospray Ionization Mass Spectrometry (*UPLC–ESI-qTOF-MS/MS*) in positive and negative ions mode analysis. Antioxidant activity was analyzed following the iron-chelating–reducing capacity and deoxyribose degradation (2-DR) protection methods. The analysis identified condensed tannins (54.8 mg catechin/g dry fraction (DF), polyphenols (25 mg gallic acid/g DF), and hydrolyzable tannins (28.8 mg tannic acid/g DF). Among the constituents, prodelphinidin, procyanidin, and prorobinetinidine were isolated and identified. The extract significantly protected 2-DR degradation induced by Fe^2+^ (72% protection) or ^•^OH (43% protection). The *ortho*-phenanthroline test revealed Fe^2+^-chelating and Fe^3+^-reducing activities of 93% and 84%, respectively. A preliminary toxicological analysis using *Artemia salina* revealed mortality below 10%, at a concentration of 0.25 mg/mL, indicating low toxicity under the present experimental conditions. In conclusion, the findings of the present study indicate that *Stryphnodendron rotundifolium* is a source of antioxidant compounds with the potential to be used in drug development in the context of iron overload disorders, which remains to be further investigated in vivo.

## 1. Introduction

*Stryphnodendron rotundifolium* Mart. is a plant species endemic to the Chapada do Araripe, Brazil, popularly known as “barbatimão”. The stem bark of this plant is widely used in traditional medicine in the preparation of tinctures, syrups, and teas for the treatment of wounds, ulcers, gastritis, inflammation, and vaginal infections [1,2].

The tannin composition in the bark of *Stryphnodendron* species ranges from 20% to 50% and is potentially responsible for its antioxidant properties [3]. In this context, previous studies have shown that alcoholic extracts obtained from the bark and leaves of *S. rotundifolium* exhibited free radical-scavenging activity and inhibited Fe^2+^-induced lipid peroxidation in brain homogenates of *Wistar* rats [4]. Accordingly, consistent evidence has demonstrated that phenolic compounds, including flavonoids and tannins, are crucially responsible for the antioxidant activity of plant-derived natural products [5,6,7].

Iron accumulation results in tissue damage by promoting the generation of reactive oxygen species (ROS), leading to the oxidation of vital molecules such as proteins, lipids, and DNA. The main clinical conditions associated with iron overload are hereditary hemochromatosis and secondary hemochromatosis. These diseases are characterized by excessive absorption of the mineral by intestinal cells, causing iron overload and tissue accumulation, which leads to organ damage, especially in the liver. Secondary hemochromatosis is commonly related to recurrent transfusion of red blood cells. In this case, iron overload is managed mainly with chelating agents [8,9,10]. Iron excess may also occur due to iron inhalation in mining and steel welding [11] and associated with transferrin deficiency [12].

With regard to iron homeostasis, the cellular mechanisms responsible for the excretion of this mineral are less developed and effective than those that regulate its absorption and distribution, which can lead to its accumulation in several iron-carrying cells [13,14].

Previous research has demonstrated that many chronic diseases result from an imbalance between ROS generation and the antioxidant mechanisms in the host cells, highlighting the importance of discovering new antioxidant products [15,16]. In addition, consistent evidence has indicated that iron overload contributes to the development of several chronic/degenerative diseases, especially cardiovascular diseases, mainly by inducing oxidative stress mechanisms, highlighting the therapeutic potential of iron-chelating agents. Therefore, the development of in vitro and in vivo assays, capable of accurately evaluating the antioxidant activity and toxicity of plant extracts, represents a significant preliminary step in the search for natural products capable of controlling iron overload disorders [17,18].

The present study reports the extraction and chemical characterization of the tannin-rich fraction of *S. rotundifolium* Mart. (TFSR). The in vitro antioxidant activity of this fraction was investigated under iron and free-radical overload conditions, and its cytotoxicity was assessed using an *Artemia salina* model.

## 2. Materials and Methods

### 2.1. Botanical Material and Extraction Procedure

*Stryphnodendron rotundifolium* stem bark samples were collected in the Araripe National Forest, in the municipality of Crato, Ceará, Brazil (coordinates: 07°81’ S, 039°28’ W). Dr. Maria Arlene Pessoa identified the species, and a voucher specimen was prepared and registered at the Herbarium of the Regional University of Cariri (registry number: 14,074).

A total of 2100 g of freshly collected barks were dried, crushed, and subjected to fat removal with hexane. Then, hexane was removed from the solid material at 70 °C. Following this process, the material was subjected to exhaustive maceration using a 7:3 acetone–water mixture for 72 h at room temperature, to avoid interactions between tannins and vegetable proteins, favoring the obtainment of more stable tannins. This procedure was repeated three times as previously described [19,20]. The resulting solution was subjected to exhaustive removal of acetone in a rotary evaporator (at 65 °C and pressure), reducing the volume to one-third of the initial extractive mixture, after which it was concentrated in a water bath at 70 °C for 24 h. The acetone-free concentrated liquid was frozen and subjected to lyophilization, after which the tannic fraction was obtained as an amorphous solid.

### 2.2. Phytochemical Prospecting

Phytochemical analysis was carried out to determine the qualitative composition of secondary metabolites. The presence of phenols, tannins, flavonoids, and alkaloids was analyzed according to a previously described method [21]. According to this method, colorimetric changes or precipitate formation in the fraction solution after the addition of specific reagents are indicative of the presence of the corresponding class of secondary metabolites. The screening of phenolic compounds was performed using FeCl_3,_ 1% methanolic solution, and 1 N HCl, 10% (*w*/*v*) NaOH, while the screening of alkaloids was performed using 5% (*v*/*v*) acetic acid, 10% (*v*/*v*) NH_4_OH, chloroform p.a., and Dragendorff’s reagent.

### 2.3. Quantification of Phenolic Compounds

#### 2.3.1. Total Phenolic Content

The total content of phenolic compounds was determined using a method developed by [22]. A total of 200 μL of the fraction aqueous solution (0.1 μg/mL) was added with 600 μL of ethanol (70% *v*/*v*) and 400 μL of the Folin–Ciocâlteu reagent (10% *v*/*v*), and the solution was vigorously shaken. After 5 min, the solution was added with Na_2_CO_3_ (2800 μL, 7.5% *m*/*v*) and incubated for 20 min at 45 °C in the absence of light. The absorbance was measured at 735 nm using the UV/Vis spectrophotometer (T80 PG Instruments LTD, Wibtoft, Leicestershire, United Kingdom. Gallic acid (GA) was used as a positive control, and the phenolic content was determined by linear regression using calibration curve (5.0–0.5 μg/mL), performed in triplicate, and the results expressed in mg GA/g dry fraction (DF) (linear equation (*y* = 0.1351*x* + 0.0727, *R*^2^ = 0.9971).

#### 2.3.2. Determination of Total Condensed Tannins

The content of condensed tannins was determined using the vanillin assay, as described by [23], with modifications. An aliquot of 0.5 mL of the fraction diluted in water (0.5 mg/mL) was added with 3 mL of vanillin (4% *v*/*v* in methanol) followed by the addition of hydrochloric acid (1.5 mL). The mixture was shaken and kept at 20 °C for 15 min in the absence of light. The absorbance of the mixture was measured at 500 nm and compared with a calibration curve (40–5 μg/mL) of a catechin aqueous solution. The content was calculated by linear regression of the calibration curve (linear equation (*y* = 0.0176*x* + 0.1464, *R*^2^ = 0.9843)) in triplicate, and the results were expressed in mg cat/g DF.

#### 2.3.3. Quantification of Total Hydrolyzable Tannins

Hydrolyzable tannins were detected according to the method proposed by [24], with modifications. A sample (0.20 g) of the fraction was extracted with 50 mL of methanol (80%) and then centrifuged at 493× *g* for 10 min. After centrifugation, 2 mL of the fraction was diluted with distilled water and mixed with 5 mL of a KIO_3_ solution (2.5% *m*/*v*) in water. The resulting solution was heated to 30 °C for 7 min °C, and, after cooling, the absorbance was read at 550 nm. The content was calculated by linear regression through a calibration curve (linear equation (*y* = 1.9636*x* + 0.0724, *R*^2^ = 0.9818) of tannic acid (tac) (0.4–0.1 mg/mL) in triplicate, and the results were expressed in mg tac/g DF.

### 2.4. UPLC–ESI-qTOF-MS/MS

These analyses were performed using an Acquity UPLC (Waters Corporation, Milford, MA, USA) system coupled to a Xevo qTOF mass spectrometer (Q-TOF, Waters). Separations were performed on a C18 column (Waters Acquity^®^ UPLC C18; 150 mm × 2.1 mm, 1.7 μm). For metabolic fingerprinting, a 2 μL aliquot of the fraction was subjected to UPLC analysis using an exploratory gradient with a mobile phase comprising deionized water (A) and acetonitrile (B), both containing formic acid 0.1% *v*/*v*. The sample was subjected to the exploratory gradient as follows: 2–95% for 15 min, at a flow rate of 500 µL·min^−1^. Ionization was performed with electrospray ionization (ESI) source in negative and positive ion modes, in the range of 110–1200 Da. The optimized instrumental parameters were as follows: capillary voltage of −2800 V, cone voltage of −40 V, source temperature of 120 °C, desolvation temperature of 330 °C, flow cone gas of 20 L·h^−1^, desolvation gas flow at 600 L·h^−1^, and microchannel plate (MCP) detector voltage of −1900 V. The mode of acquisition was MS/MS, and the system was controlled using MassLynx 4.1 software (Waters Corporation, Milford, MA, USA).

### 2.5. In Vitro Antioxidant Activity Analysis

#### 2.5.1. Fe^2+^-Chelating Activity and Fe^3+^-Reducing Power as a Function of Time

In these experiments, the protective activity of the fraction was evaluated in reactive mixtures containing Fe^2+^ at the concentration of 100 μM, which is equivalent to 5.58 μg/mL. Since normal iron concentration in the blood ranges from 0.65 μg/mL to 1.7 μg/mL and the maximum concentration of iron transported by transferrin (tf) in blood is in the range of 2.5 μg/mL to 4.5 μg/mL [25,26,27,28], the protocol follow was used as an in vitro model of Fe^2+^ overload.

The Fe^2+-^chelating and Fe^3+-^reducing capacity of the fraction were evaluated through the *o*-phenanthroline (*o*-phe) assay, as described by the method of [29], with some modifications. Samples were separately added with Fe^2+^ and Fe^3+^ through the addition of FeSO_4_ and FeCl_3_ (1 mM), respectively, in a dark and refrigerated environment. Then, aliquots of 40 μL were extracted at times 0.5, 2.5, 5, 10, 20, 30, 45, 60, 75, and 90 min and reacted with *o*-phe (300 μM) in H_2_O milli-Q and Tris-HCl (0.1 M, pH 7.4). At the end of this procedure, the samples were obtained at final concentrations of 0.2, 0.1, and 0.05 mg/mL, and Fe^2+^ and Fe^3+^ were obtained both at 100 μM. The readings were carried out at 510 nm. Experimental controls were obtained by replacing aliquots of the samples with milli-Q water. To evaluate the stability of the chelates, ascorbic acid (at the final concentration of 0.005 M) was added to each reacting system (at the timepoint of 90 min), and the readings were performed 15 min later. The results are representative of three independent experiments (*n* = 3) performed in duplicate.

The Fe^2+^-chelating activity (FCA) and Fe^3+^-reducing power (FRP) were expressed as a percentage of the control, as represented by the Equations (1) and (2), respectively.

(1) $$\mathrm{FCA}\left(\%\right)t_{\mathrm{minutes}}=\frac{\left({\mathrm{Abs}}_{\mathrm{controle}-{\mathrm{Fe}}^{2+}}-\left({\mathrm{Abs}}_{\mathrm{fraction}-{\mathrm{Fe}}^{2+}}-{\mathrm{Abs}}_{\mathrm{fraction}-\mathrm{blank}-{\mathrm{Fe}}^{2+}}\right)\right)\times 100}{{\mathrm{Abs}}_{\mathrm{controle}-{\mathrm{Fe}}^{2+}}}$$

(2) $$\mathrm{FRP}\left(\%\right)t_{\mathrm{minutes}}=\frac{\left({\mathrm{Abs}}_{\mathrm{fraction}-{\mathrm{Fe}}^{3+}}-{\mathrm{Abs}}_{\mathrm{fraction}-\mathrm{blank}-{\mathrm{Fe}}^{3+}}\right)-{\mathrm{Abs}}_{\mathrm{control}-{\mathrm{Fe}}^{3+}})\times 100}{{\mathrm{Abs}}_{\mathrm{controle}-{\mathrm{Fe}}^{2+}}}$$

where Abs_Control-Fe^2+^_ is the absorbance of *o*-phe + Fe^2+^, Abs_Fraction-Fe^2+^_ is the absorbance of fraction + *o*-phe + Fe^2+^, Abs_Fraction-blank-Fe^2+^_ is the absorbance of fraction + Fe^2+^, Abs_Control-Fe^3+^_ is the absorbance of *o*-phe + Fe^3+^, Abs_Fraction-Fe^3+^_ is the absorbance of fraction + *o*-phe + Fe^3+^, and Abs_Fraction-blank-Fe^3+^_ is the absorbance of fraction + Fe^3+^.

#### 2.5.2. Deoxyribose Oxidative Degradation Assay

The ^•^OH radical-scavenging activity was investigated according to the ability of the fraction to inhibit the degradation of 2-deoxyribose (2-DR) in vitro, following the method used by [30], with adaptations. Briefly, 1.5 mM 2-DR was incubated with 50 mM potassium phosphate buffer (pH 7.4) at room temperature for 20 min in the following pro-oxidant systems: (i) 0.05 mM FeSO_4_ and (ii) 0.5 mM H_2_O_2_ + 0.05 mM FeSO_4_ (Fenton reaction); then, it was incubated at 37 °C for 60 min, in the absence or presence of the variable concentrations of the fractions (0.2, 0.1, and 0.075 mg/mL). Following this step, 750 μL of Trichloroacetic Acid (TCA) (2.8%) and 750 μL of Thiobarbituric Acid (TBA) (0.8%) were added to all samples, before incubating for 20 min at 100 °C. The readings were carried out at 532 nm, and the results are expressed as a percentage of inhibition of 2-DR degradation relative to the negative controls according to Equation (3).

(3) $$\mathrm{Inhibition}\left(\%\right)=\frac{\left[\left({\mathrm{Abs}}_{\mathrm{control}}-{\mathrm{Abs}}_{\mathrm{blank}}\right)\right]\times 100\%}{{\mathrm{Abs}}_{\mathrm{control}}}$$

where Abs_Control_ is the Absorbance of 2-DR + pro-oxidant, Abs_Fraction_ is the absorbance of 2-DR + pro-oxidant + fraction, and Abs_Blank_ is the absorbance of pro-oxidant + fraction.

Of note, the Fenton reaction was performed with 50 μM Fe^2+^, which is equivalent to 2.79 μg/mL. The results are representative of four independent experiments (*n* = 4) performed in duplicate.

#### 2.5.3. Analysis of the Mechanism Underlying the Inhibition of 2-DR Degradation

An assay based on the variation of the 2-DR concentration (1.5 mM, 1.75 mM, and 2.0 mM) was used to determine the antioxidant mechanism of the fraction (Fe^2+^ chelating or ^•^OH scavenging). The oxidative degradation of 2-DR was obtained via the reaction of 0.5 mM H_2_O_2_ with 0.05 mM FeSO_4_, which results in the generation of the ^•^OH radical. The tests were performed three (*n* = 3) times in duplicate for each concentration of 2-DR (1.5 mM, 1.75, mM, and 2.0 mM). The results were expressed as a percentage inhibition of 2-DR degradation according to Equation (4) [31].

(4) $$\mathrm{Inhibition}\left(\%\right)=\frac{[{\mathrm{Abs}}_{\left(H2O2+{\mathrm{Fe}}^{2+}\right)\mathrm{control}}-\left({\mathrm{Abs}}_{\mathrm{fraction}}-{\mathrm{Abs}}_{\mathrm{blank}}\right)]\times 100\%}{{\mathrm{Abs}}_{\left(H2O2+{\mathrm{Fe}}^{2+}\right)\mathrm{control}}}$$

where Abs_(H_2_O_2_ + Fe^2+^)Control_ is the absorbance of 2-DR + (H_2_O_2_ + Fe^2+^), Abs_Fraction_ is the absorbance of 2-DR + (H_2_O_2_ + Fe^2+^) + fraction, and Abs_Blank_ is the absorbance of (H_2_O_2_ + Fe^2+^) + fraction.

### 2.6. In Vivo Toxicity Analysis

Saline solution (36.2 g/L, pH 7.0–9.0) was prepared by adding 72.4 g of sea salt in 2 L of distilled water. A total of 200 mg of *Artemia Salina* eggs were placed to hatch in 400 mL of saline solution for 24 h under continuous aeration and exposure to light. To determine the in vivo toxicity of the fraction, 10 larvae were placed in 10 mL of a solution containing the fraction at increasing concentrations (0.001 to 0.250 mg/mL). The mortality was analyzed 24 h after treatments. The tests were performed in triplicate [32].

### 2.7. Statistical Analysis

The data of the antioxidant and toxicity assays, as well as those of chemical quantification, were expressed as the means ± Standard Error of Mean (SEM) of at least three experiments. The antioxidant assays were analyzed by one-way analysis of variance (ANOVA), followed by Tukey’s post hoc test for multiple comparisons of data with normal distribution and similar standard deviation, using GraphPad Prism version 6.0. The UPLC/MS data were expressed as the means ± SEM of three determinations and analyzed by ANOVA followed by Tukey’s test. Statistical significance was considered when *p* < 0.05.

## 3. Results and Discussion

The tannin-rich fraction from *Stryphnodendron rotundifolium* Mart. (TFSR) was obtained as a reddish solid, characteristic of proanthocyanidins. The acetone–water (7:3) extraction presented a yield of 38.8%. The phytochemical characterization demonstrated the presence of secondary metabolites such as polyphenols, flavonoids, leucoanthocyanidins, aurones, chalcones, catechins, and hydrolyzable and condensed tannins. In particular, the UPLC–ESI-qTOF-MS/MS profile revealed the presence of type B proanthocyanidins, including prodelphinidins (monomers, dimers, and trimers), procyanidins, and prorobinetidins previously reported in other species of the same genus [5,33,34].

The concentration of total phenols was determined in 25.02 ± 0.67 mg gallic acid/g DF. The quantification of condensed tannins was 54.83 ± 2.30 mg catechin equivalent/g DF, corresponding to the main type of component in the sample. In addition, the concentration of hydrolyzable tannins was 28.84 ± 2.21 mg tannic acid/g DF, confirming this class of components as the second most abundant in the sample. In fact, extractive mixtures containing acetone favor the extraction of condensed tannins formed by monomeric units of the catechin type flavan-3-ols, to the detriment of hydrolyzable tannins [19].

**Figure 1:**
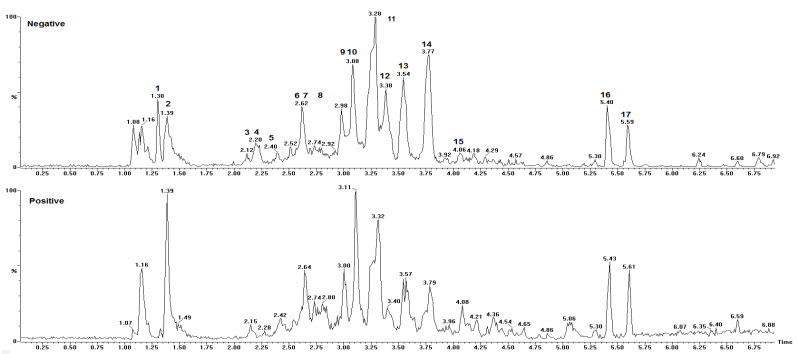
Chromatogram: assignment of peaks to the metabolites in the acetone–water fraction from the stem bark of *S. rotundifolium* Mart. using Ultra-Performance Liquid Chromatography coupled with a quadrupole-Time Of Flight, with Electrospray Ionization Mass Spectrometry (UPLC–ESI-qTOF-MS/MS) in the negative and positive modes. The monitored ions correspond to the most abundant protonated and deprotonated molecules, using a restricted window of 0.0001 *m*/*z* units centered on each selected ion. Peak 2: 4’-*O*-methyl-epigallocatechin (4→8) epigallocatechin; peak 3: epigallocatechin (4β→8) epigallocatechin; peak 4: gallic acid; peak 5: epigallocatechin (4→8) epigallocatechin (4→ 8) epigallocatechin; peak 6: 4’-*O*-methyl-epigallocatechin-3-*O*-gallate (4→6) epigallocatechin 3-*O*-gallate; peak 7: epigallocatechin (4β→6) epigallocatechin; peak 8: epigallocatechin (4→8) epigallocatechin (4→8) epigallocatechin-3-*O*-gallate; peak 9: epigallocatechin; peak 10: epigallocatechin (4→8) epigallocatechin-3-*O*-gallate/epigallocatechin-3-*O*-gallate (4→8) epigallocatechin; peak 11: robinetinidol-4’-*O*-methyl (4→8) epigallocatechin; peak 12: C-hexosyl-*O*-pentosyl-5,7-dihydroxychromone isomer; peak 13: procyanidin prodelphinidin type B.

The chemical constituents of the TFSR were identified using the UPLC–ESI-qTOF-MS/MS technique, by interpreting their MS and MS/MS spectra, determined by QTOF-MS and compared with the literature data and the open-access mass spectrum database SciFinder. The chromatogram and phenolic profile are shown in Figure 1 and Table 1, respectively.

A total of 17 peaks were identified, revealing the presence of compounds belonging to corresponding to two main classes of metabolites: phenolic acids and tannins. The compounds were eluted in a noticeably short period of time (6.9 min). Together, the retention time and the number of peaks observed in the present analysis indicate that UPLC is an efficient separation technique.

**Figure 2:**
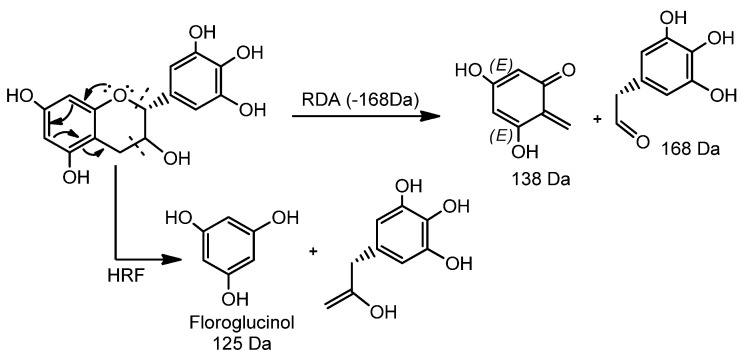
Mechanistic pathways of RDA reactions and heterocyclic cleavage of the Heterocyclic Fission (HRF) C ring.

According to our analysis, peaks 4 and 9 exhibited protonated ions in *m*/*z* 171.0300 and 307.0824, respectively, which is compatible with the corresponding molecular formulas C_7_H_6_O_5_ and C_15_H_14_O_7_, as their fragmentation profiles are compatible with the reported for *S. adstringens* [5,33,35]. The fragmentation pattern id compatible with the mechanistic pathway of retro-Diels–Alder (RDA) reactions and the different heterocyclic cleavage of the C ring (HRF—heterocyclic fission) (Figure 2). In addition, the occurrence of dehydration was crucial for the identification of peak 9 epigallocatechin (EGC) [37].

The protonated ions at *m*/*z* 625.5280 (peak 2), 611.1428 (peak 3), 929.7240 (peak 6), 611.1406 (peak 7), 763.1538 (peak 10), 609.5290 (peak 11), and 595.1460 (peak 13), corresponding respectively to C_31_H_28_O_14_, C_30_H_26_O_14_, C_45_H_36_O_22_, C_30_H_68_O_14_, C_37_H_30_O_18_, C_31_H_28_O_13_, and C_30_H_26_O_13_, were characterized as type B dimeric proanthocyanidins and type B dimeric proanthocyanidins with 3-*O*-galloyl in epigallocatechin (EGC) subunits.

The spectral analysis of peaks 2, 3–8, 10, 11, and 13 revealed fragmentation ions at *m*/*z* 319, 307, 305, 289, and 287, produced from the generation of quinone and quinone methide with losses of (110, 125, 181, 318) *u*, 289 *u*, (319, 457, 471, 153) *u*, 289 *u*, (305, 609, 761) *u*, and H_2_O molecules, consistent with prodelphinidin (PDE), procyanidin (PCY), and prorobinetinidine (PRO). Thus, the common losses due to RDA reactions of 168 and 152 Da, as well as the heterocyclic cleavage reactions of the C HRF 125 Da ring (floroglucinol), suggest the presence of PDE, PRO, and PCY.

Pseudomolecular ion products at *m*/*z* 425 [M + H^+^ − 168 − H_2_O]^+^, [M + 2H^+^ − 305 − 168 − H_2_O]^+^, [M + 2H^+^ − 457 − 168 − H_2_O]^+^ resulting from RDA reactions and excisions of 305 EGC and 457 gallate epigallocatechin (EGCG) were observed for peaks 3, 5, 7, and 8. An ion at *m*/*z* 427 [M + H^+^ − 168]^+^ was observed for metabolite 13. The ions at *m*/*z* 485 [M + H^+^ − 153 − 125]^+^ and [M + 2H^+^ − 125]^+^ resulted from the excisions of the galloyl (153 Da) and floroglucinol (125 Da) groups, respectively, corresponding to peaks 10 and 11. Of note, six possible isomers were proposed for peak 10. The ion at *m*/*z* 423 [M + 2H^+^ − 169 − 168 − 170]^+^ was associated with peak 6, such that the 169 Da fragment was associated with the loss of an O-gallate group, in which the 168 Da fragment corresponds to the loss of the 4-*O*′-methyl-3,5-di-hydroxyl-benzaldehyde portion through heterocyclic cleavage of the C ring. The 170 Da fragment was associated with the loss of a gallic acid unit. Together, these findings suggest the presence of dimeric/trimeric PDE, in addition to PCY and dimeric PRO in the phenolic fraction under investigation, whose spectral profiles are compatible with the literature data [5,7,33,34,35].

The type B prodelphinidin trimer shown in peak 5 was identified as three EGC units exhibiting the protonated ion at *m*/*z* 915.2008, which corresponds to C_45_H_38_O_21_, as also reported in *S. adstringens* [5]. On the other hand, the type B prodelphinidin trimer in peak 8, consisting of two units of EGC and one EGCG unit that exhibited the protonated ion at *m*/*z* 1066.2070, corresponds to C_42_H_42_O_25_, as previously reported in *Myrica yubra* Sieb & Zucc [7]. Of note, proanthocyanidin ions larger than trimers were not identified, since these experiments were recorded in a 110–1200 Da *m*/*z* window.

The characteristic of peak 12 is compatible with the structure of chromonic compounds [38]. Accordingly, its protonated ion presented a precise mass loss at *m*/*z* 487.1447, corresponding to C_21_H_26_O_13_. The fragmentation of ions at *m*/*z* 487 produced an ion at *m*/*z* 355 [M + H − 132]^+^ suggesting an *O*-pentosyl substitute in the structure, at *m*/*z* 319 [M + H − 168]^+^. Thus, peak 12 was identified as C-hexosyl O-pentosyl 5, 7-dihydroxychromone, and its spectral data are comparable to the data reported in the literature [5].

In synthesis, the UPLC–MS analysis showed the predominance of type-B proanthocyanidins (PA) with C_4_→C_8_ and C_4_→C_6_ interflavanic bonds in the tannin-rich fraction of *S. rotundifolium.*

**Figure 3:**
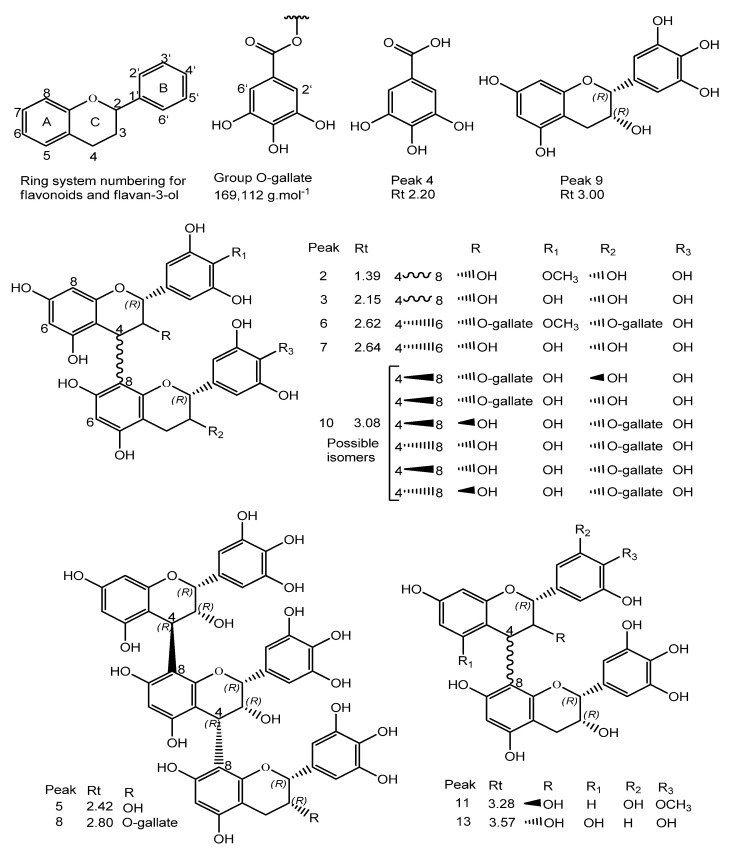
Molecular structures of the metabolites identified in the tannic fraction of *Stryphnodendron rotundifolium* Mart., (TFSF).

The molecular structures of compounds (Figure 3) were designed using the Chem Draw Ultra 12.0.2.1076.lnk program (Licensed to Mulder X-Filer 875-317589-4732. 1986–2010 CambridgeSol).

The findings of the present study indicate that the phenolic composition of *S. rotundifolium* is characterized by a remarkable presence of type B proanthocyanidins, a group of compounds with demonstrated antioxidant activity, which may, therefore, play important roles in health maintenance and minimization of disease onset and progression [2,39].

The Fe^2+^-chelating activity and Fe^3+^-reducing power of TFSR, as a function of time and concentration, were analyzed using the *o*-phe assay. As shown in Table 2, the fraction was effective in chelating Fe^2+^ at all concentrations and timepoints evaluated in this study. At the highest concentration (0.200 mg/mL), the activity varied over time (0.5 to 90 min), reaching an average Fe^2+^-chelating rate of 93%. For the lowest concentration (0.05 mg/mL), an average value of 77% was found. The fraction, at the concentrations analyzed, reached maximum activity at 45 min. Importantly, there was no significant difference in the chelating activity at different timepoints when considering the same concentration. However, a significant difference was observed between the Fe^2+^-chelating activity of the concentrations 0.200 mg/mL and 0.050 mg/mL. The results also indicate the chelating activity of the fraction is not concentration-dependent.

The rapid decrease in the absorbance of the Fe^2+^/*o*-phe/TFSR system compared to the control (*o*-phe/Fe^2+^/H_2_O) indicates that the formation of chelates with Fe^2+^ is kinetically favored. The addition of ascorbic acid 90 min after the beginning of the reaction caused insignificant variation in the absorbance of the sample, revealing the generation of chelates with higher thermodynamic stability then the ferroin chelate (ph)_3_Fe^2+^.

A study by [36] reported that the Fe^2+^ chelating potential of the TFSR was positively correlated with the number of catechol groups in the phenolic structures. Therefore, the abundance of highly hydroxylated proanthocyanidins with catechol and galloyl groups in the tannin-rich fraction under study offer several chelation sites, contributing to its significant Fe^2+^-chelating activity.

Regarding the Fe^2+^-chelating activity of *S. rotundifolium*, this work demonstrated that its tannin-rich fraction showed a potent effect, with fast reaction kinetics. The reaction profile indicates that the chelates formed between the fraction constituents and Fe^2+^ are thermodynamically more stable than the ferroin chelate of high crystal field stabilization energy (CFSE) [40]. Consequently, the authors of this work believe that the significant chelating activity of the fraction is related to synergistic effects due to the presence of prodelphinidins, prorobinetidine, and procyanidin with many catechol and galloyl groups. The potent iron-chelating activity exhibited by *S. rotundifolium* indicates that this species has the potential to be used to minimize oxidative effects of iron overload eating conditions that play important pathological functions, including a wide variety of disorders such as tumors, nervous system diseases, ischemia/reperfusion injury, kidney injury, and blood diseases [41].

As for the Fe^3+^-reducing power, at the highest concentration analyzed (0.200 mg/mL), the fraction effectively reduced Fe^3+^ to Fe^2+^ from the first timepoint of analysis (Table 3), exhibiting strong reducing activity with an average of 84% (from 0.5 to 90 min) and the peak effect reached at 45 minutes, revealing the typical electron transfer characteristic of phenolic compounds [42]. Nevertheless, in this assay, the maximum activity of the fraction from 0.20 to 0.05 mg/mL was reached at 105 min. The rapid decrease in the absorbance of the systems (Fe^3+^/*o*-phe/TFSR) in relation to the control (*o*-phe/Fe^2+^/H_2_O), suggests that the reducing reaction from Fe^3+^ to Fe^2+^ is kinetically favored in the presence of the constituents of the fraction. The addition of ascorbic acid 90 min after the beginning of the reaction caused a decrease in the absorbance of the system (Fe^3+^/*o*-phe/TFSR), revealing that the Fe^2+^ fraction was subtracted from the ferroin chelate by the action of the TFSR, reducing the absorbance of ferroin. As observed in the iron-chelating activity experiment, no significant difference was observed with regard to the reducing activity when different timepoints at the same concentration were compared. However, there was a significant difference in Fe^3+^-reducing activity between the concentration of 0.200 mg/mL and the others (0.100 mg/mL and 0.050 mg/mL). The results obtained in this experiment indicate that the reducing activity responses mediated by the extract do not occur in a concentration-dependent manner.

The ^•^OH radical-scavenging activity was evaluated using the 2-deoxyribose (2-DR) oxidative degradation method, by quantifying its main subproduct, malondialdehyde (MDA). The antioxidant activity was expressed as the percent protection of 2-DR degradation.

The hydroxyl radical (^•^OH) is a highly reactive free radical capable of triggering damage to several cellular structures. *In vivo*, this radical can be generated in the presence of superoxide radicals and transition cations, such as iron and copper, through the Haber–Weiss reaction [43]. In the experimental conditions of this study, the ^•^OH radical was generated in vitro through the Fenton reaction.

**Figure 4:**
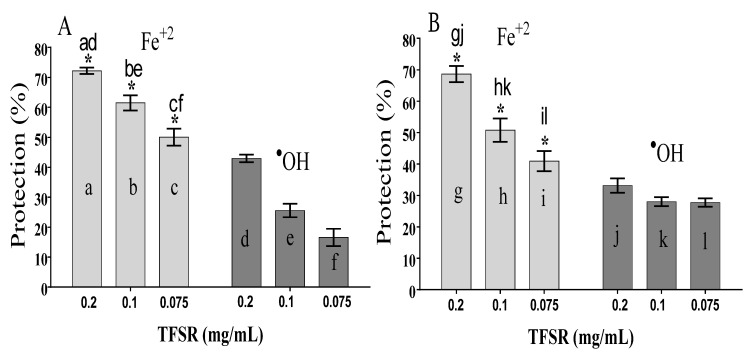
Percentage (%) protection of 2-DR degradation in vitro. The antioxidant activity of the tannin-rich fraction of *S. rotundifolium* (TFSR) was evaluated previously (**A**) or simultaneously (**B**) to the addition of Fe^2+^ (50 μM, light bars) or the Fenton reaction reagents (50 μM Fe^2+^ + 0.5 mM H_2_O_2,_ dark bars). These values were expressed as the means ± SEM (*n* = 4) and analyzed by one-way ANOVA followed by Tukey’s multiple comparison test, with a simple pooled variance. * Significant difference (*p* < 0.05) at equal concentrations. a–l, represent the concentrations of TFSR in the different pro-oxidant systems.

Figure 4A,B show the protection level exerted by the fraction when the treatment was performed previously (PT) or simultaneously (ST) to the addition of either of the prooxidant systems: Fe^2+^ (light-gray bars) and Fenton reaction/^•^OH (dark-gray bars). In both treatment conditions, the fraction showed a strong protective activity against the oxidative action of Fe^2+^ and a moderate activity against the action of the ^•^OH radicals generated in the Fenton reaction. For the first condition, the fraction presented IC_50_ values of 0.0576 and 0.100 mg/mL for PT and ST, respectively. For the second condition, these values were respectively 0.225 and 0.596 mg/mL. However, considering the distinct pro-oxidant systems, no significant difference in the protective activity of the fraction against 2-DR degradation for a given concentration was observed, indicating that the antioxidant activity was not influenced by the time of treatment.

At the concentration of 0.2 mg/mL, the TFSR prevented 2-DR degradation by the pro-oxidant Fe^2+^ with protection levels of 72.2% and 68.6% for PT (Figure 4A) and ST (Figure 4B), respectively. However, the difference between these treatment conditions was not significant. The 2-DR degradation triggered by the ^•^OH radical generated in the Fenton reaction was inhibited by TFSR (0.2 mg/mL) in both PT (Figure 4A) and ST (Figure 4B) conditions, with protection levels of 42.9% and 33.1%. Again, the difference between these treatment conditions was not significant.

Considering the two different oxidant systems, it can be observed that TFSR protective activity against the pro-oxidant condition Fe^2+^ was significantly higher that observed against ^•^OH radical produced in the Fenton reaction, as shown in Figure 4.

The remarkable ^•^OH-scavenging activity of the fraction can be attributed to the presence of hydrolyzable tannins, especially type B proanthocyanidins (dimers and trimers) with catechol and pyrogallol groups. These compounds have been recognized as excellent natural antioxidants and, as such, have the potential to be investigated in pharmacological research in several disease models [44]. Studies have indicated that the mechanism underlying the antioxidant activity of these compounds involves the donation of hydrogen and electrons responsible for the inhibition of free radicals [5,7].

To elucidate the mechanism via which the fraction protects 2-DR from degradation in the presence of the Fenton reaction, we evaluated the protective effect of different concentrations of the fraction (0.2, 0.1, and 0.075 mg/mL) using varying concentrations of 2-DR (1.5, 1.75 and 2.0 mM) and a fixed concentration of the reacting system (50 μM Fe^2+^ + 0.5 mM H_2_O_2_).

The protection levels exerted by different concentrations of TFSR against 2-DR degradation did not vary significantly following the increasing 2-DR concentrations (1.5, 1.75, and 2.0 mM) (Table 4), suggesting that the constituents of the fraction act predominantly as iron-chelating antioxidants, inhibiting the generation ^•^OH radicals.

The findings of the present research indicate that the protective mechanism of TFSR occurred mainly via chelation of Fe^2+^ ions (as show in Table 4) and, to a lesser extent, via ^•^OH radical scavenging. Importantly, this mechanism can prevent Fe^2+^ from participating in reactions that generate free radicals such as H_2_O_2_ and ^•^OH, which could have beneficial roles in inhibiting oxidative stress. Nevertheless, a secondary antioxidant mechanism via ^•^OH scavenging could contribute to the occurrence of synergistic antioxidant effects.

**Figure 5:**
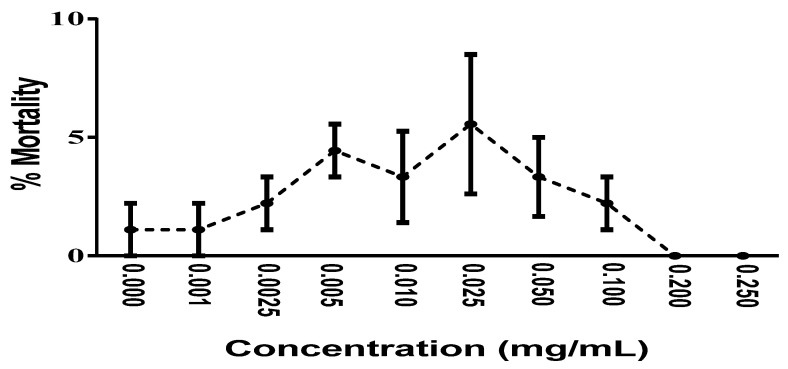
*Artemia salina* toxicity of the acetone–water fraction obtained from the stem bark of *Stryphnodendron rotundifolium* Mart. Mortality was analyzed 24 h after the addition of different fraction concentrations. These values are expressed as the means ± SEM (*n* = 3), with a 95% confidence interval and analyzed by nonlinear regression of the transformed curves using one-way ANOVA followed by Bonferroni correction and Tukey’s multiple comparison test, with a simple pooled variance.

The preliminary toxicological analysis using *A. salina* showed that, in the range of concentrations evaluated (0.001 to 0.25 mg/mL), mortality below 10% was observed, with no statistical difference between the rates of mortality induced by each of the tested concentrations, indicating that the fraction has low in vivo toxicity under the experimental conditions adopted in the present study (Figure 5).

The results demonstrated by this preliminary toxicological evaluation suggest that the fraction can be tested in a higher concentration range, both in pharmacological and in toxicological studies, since no evident toxicity was observed at the highest tested concentration (0.25 mg/mL). However, additional studies using cell cultures and animal models are fundamental to bring relevant information regarding its in vivo safety, especially with regard to the risks associated with the ingestion of tannins.

It is hypothesized that the antioxidant effects demonstrated by the fraction are explained, at least partially, by the presence of constituents carrying pyrogallol groups containing hydroxyls capable of promoting both Fe^3+^ reduction and Fe^2+^ chelation, in addition to potentially acting as electron and hydrogen donors and, as such, acting as free-radical scavengers. The presence of galloyl portions, as well as the stabilization of phenoxyl radicals by resonance, contributes significantly to the antioxidant capacity of phenolic compounds [45]. In addition, the existence of significant amounts of galloyl portions in polyphenols favors the stabilization of phenoxyl free radicals (semiquinones), contributing to their iron-chelating and radical-scavenging activities [46].

In this context, the proanthocyanidins identified in the tannin-rich fraction present characteristics that favor their antioxidant action. In addition, these compounds can be easily absorbed by the gastrointestinal tract, which is a desirable characteristic for substances used in the development of oral drugs. Notwithstanding, proanthocyanidins with higher polymerization rates have low permeability coefficients in the human organism, impairing their oral absorption [47].

Despite the strong influence of pyrogallol and phenolic hydroxyl groups on TFSR antioxidant activity, the three-dimensional arrangement of the structure and possibly steric effect might contribute to the pharmacological effects of the fraction components. Thus, among the proanthocyanidins identified, we believe that dimers and trimers mostly contribute to the antioxidant activity demonstrated through 2-DR degradation protection.

Iron is a mineral with several physiological functions, found both in free form and in the composition of proteins such as hemoglobin, myoglobin, cytochromes, and several enzymes [48]. However, due its deleterious effects, the cells store free iron at low levels.

While little amounts of this mineral are commonly stored by macrophages, excess free iron reaches the circulation and deposits in hepatocytes and parenchymal cells. Iron accumulation triggers oxidative stress by mechanisms that involve the Fenton and Haber–Weiss reactions. In this phenomenon, free iron acts as a catalyst for oxidative reactions stimulating the synthesis of superoxide and hydroxyl radicals [49].

Recent research has demonstrated that many of the deleterious effects of iron occur due to ferroptosis, an iron-dependent type of cell death characterized by the accumulation of lipid ROS that play important roles in a wide variety of systemic diseases, such as nervous system diseases, heart diseases, liver diseases, gastrointestinal diseases, lung diseases, kidney diseases, and pancreatic diseases [42]. Hence, the importance of demonstrating the expressive ability of TFSSR to acting as iron-chelating–reducing in vitro raises the hypothesis that this natural product may present promising results in vivo, which will be further investigated.

In fact, evidence suggests that substances capable of forming complexes with iron, thus promoting its excretion, may have applications in the treatment of several conditions [50], highlighting the therapeutic potential of *S. rotundifolium,* which proved to be a source of tannic compounds with Fe^2+^-chelating and ^•^OH-scavenging activities. This hypothesis is supported by studies demonstrating that extracts with high concentrations of phenolic compounds inhibited lipid peroxidation in human erythrocytes by decreasing the production of malondialdehyde, a subproduct of 2-DR degradation [51].

In recent years, considerable interest has arisen regarding the beneficial effects of proanthocyanidins (PAs) and their monomers on human health. Studies have shown that these substances have numerous pharmacological activities, including antioxidant, anti-inflammatory, and anticancer properties [5,39,52]. Therefore, the polymeric proanthocyanidins identified in *S. rotundifolium* could have beneficial effects in metabolic disorders, preventing the onset of diseases whose etiology is associated with iron overload and oxidative stress [53].

## 4. Conclusions

The results of the present study demonstrated that the tannin-rich fraction of *S. rotundifolium* is a natural product with significant in vitro antioxidant activity, which may be directly related to the high content of prodelphinidins, prorobinetidines, and highly hydroxylated procyanidins with great capacity to donate electrons, as well as to chelate iron ions.

The potent Fe^2+^-chelating activity demonstrated by the tannin-rich fraction of *S. rotundifolium* and the significant and constant protective activity observed in the 2-DR + Fe^2+^ system indicate that the antioxidant mechanisms of this fraction are mainly via an iron-chelating action and, to a lesser extent, an ^•^OH radical-scavenging action, at concentrations that do not cause significant toxicity in vivo in the *Artemia salina* model.

In conclusion, the tannic fraction of *S. rotundifolium* Mart. has potent antioxidant and iron-chelating activities, which indicates that it is a source of antioxidant compounds with the potential to be used in drug development in the context of iron overload disorders, which remains to be further investigated in vivo.

## Figures and Tables

**Table 1:** Identification of TFSR phenolic compounds using UPLC–ESI-qTOF-MS/MS in the negative and positive modes.

Peak No.	Rt (min)	[M + H]^+^ Obs	[M − H]^−^ Obs	Empirical Formula [M − H]^−^	Ion Products (MS/MS)	Empirical Formula [M + H]^+^	Empirical Formula M/MM (g·mol^−1^)	∆ ^1^ (Error)	Structure Name	Ref.
1	1.30	383.1159	no ^2^	-	203.0517	C_21_H_19_O_7_		−2.3	Unknown	-
2	1.39	625.5280	623.1370	C_31_H_27_O_14_	125.0226, 169.0148, 305.0661	C_31_H_29_O_14_	C_31_H_28_O_14_/624.5603	3.7	4’-*O*-methyl-epigallocatechin (4→8) epigallocatechin	[33]
3	2.15	611.1428	609.1245	C_30_H_25_O_14_	425.0808, 299.0632, 287.0573, 263.0517, 179.0361	C_30_H_27_O_14_	C_30_H_26_O_14_/610.494	4.4	Epigallocatechin (4β→8) epigallocatechin	[5,33,34,35]
4	2.20	171.0300	169.0138	C_7_H_5_O_5_	-	C_7_H_7_O_5_	C_7_H_6_O_5_/170.113	4.1	Gallic acid ^3^	[5,35,36]
5	2.42	915.2008	913.1827	C_45_H_37_O_21_	611.1431, 425.0869, 287.0597, 263.0562, 179.0362	C_45_H_39_O_21_	C_45_H_38_O_21_/914.733	2.6	Epigallocatechin (4→8) epigallocatechin (4→8) epigallocatechin	[5]
6	2.62	929.7240	927.2041	C_45_H_35_O_22_	761.1112, 423.0788, 305.0630, 125.0252	-	C_45_H_36_O_22_/928.311	0.2	4’-*O*-methyl-epigallocatechin-3-*O*-gallate (4→6) epigallocatechin 3-*O*-gallate	[33]
7	2.64	611.1406	609.1224	C_30_H_25_O_14_	425.0943, 287.0575, 263.0571, 179.0337	C_30_H_27_O_14_	C_30_H_26_O_14_/610.494	0.8	Epigallocatechin (4β→6) epigallocatechin	[5,34,35]
8	2.80	1067.2070	1065.1937	C_52_H_41_O_25_	915.2032, 611.1549, 425.0953, 287.0670, 263.0620, 179.0453	C_52_H_43_O_25_	C_52_H_42_O_25_/1066.831	−2.2	Epigallocatechin (4→8) epigallocatechin (4→8) epigallocatechin-3-*O*-gallate	[7]
9	3.00	307.0824	305.0664	C_15_H_13_O_7_	195.0573, 177.0488, 163.0406	C_15_H_15_O_7_	C_15_H_14_O_7_/306.255	2.0	Epigallocatechin	[5,33,35]
10	3.08	763.1538	761.1367	C_37_H_29_O_18_	593.1270, 485.1284, 319.0786, 305.0662, 125.0253	C_37_H_31_O_18_	C_37_H_30_O_18_/762.6221	1.7	Epigallocatechin (4→8) epigallocatechin-3-*O*-gallate/Epigallocatechin-3-*O*-gallate (4→8) epigallocatechin	[7,33,34,35]
11	3.28	609.5290	607.1412	C_31_H_27_O_13_	485.1264, 319.0786, 287.0568	C_31_H_29_O_13_	C_31_H_28_O_13_/608.5451	1.4	Robinetinidol-4’-*O*-methyl (4→8) epigallocatechin	[34,35]
12	3.32	487.1447	485.1295	C_21_H_25_O_13_	355.1012, 319.0728, 289.0634, 259.0592	C_21_H_27_O_13_	C_21_H_26_O_13_/ 486.405	−1.0	C-hexosyl-*O*-pentosyl-5,7-dihydroxychromone isomer	[5]
13	3.57	595.1460	593.1277	C_30_H_25_O_13_	427.0926, 307.0875, 289.0704	C_30_H_27_O_13_	C_30_H_26_O_13_/594.495	1.3	Procyanidin prodelphinidin type B	[5]
14	3.79	623.1767	no ^2^	-	423.1066, 287.0533	C_32_H_31_O_13_	-	0.3	Unknown	-
15	4.08	623.1779	no ^2^	-	423.1018, 287.0552	C_32_H_31_O_13_	-	2.2	Unknown	-
16	5.43	677.2097	no ^2^	-	545.1690, 367.1169, 235.0579, 191.0706	C_32_H_37_O_16_	-	2.2	Unknown	[5]
17	5.60	707.2196	no ^2^	-	575.1804, 367.0898, 236.0611, 221.0834	C_33_H_39_O_17_	-	1.3	Unknown	

Rt min: retention time of the eluted compounds under the experimental conditions described in the UPLC–MS section; [M − H]^−^: exact mass value of the deprotonated molecule; [M + H]^+^: exact mass value of the protonated molecule; M: exact mass value of the neutral molecule; MM: molar mass; (MS/MS): *m*/*z* mass–charge ratio of the major ions produced by the fragmentation of deprotonated molecules. ∆ ^1^ mass error (ppm) in parts per million; ppm = 10^6^ × (accurate mass exact); ^1^—positive mode; ^2^—non-observed; ^3^—compared to the standard. The signals detected in the present analysis were numbered following the elution order (retention time).

**Table 2:** Fe^2+^-chelating activity of TFSR.

[TFSR] mg/mL	(%)Fe^2+^-Chelating Activity/Time (min)										Asc. ac.
	0.5	2.5	5	10	20	30	45	60	75	90	105
0.200	87.88 ± 2.40	93.61 ± 1.78	93.29 ± 1.93	92.32 ± 1.75	95.37 ± 2.96	93.37 ± 3.29	97.82 ± 0.91	95.24 ± 2.69	93.15 ± 3.37	91.86 ± 3.08	91.94 ± 5.04
0.100	86.77 ± 4.25	84.55 ± 2.87	83.62 ± 2.62	85.77 ± 2.81	85.81 ± 1.77	84.44 ± 4.32	93.68 ± 3.47	86.32 ± 4.11	82.66 # ± 4.58	79.50 # ± 2.65	77.92 # ± 5.24
0.050	76.50 # ± 3.50	80.92 # ± 3.39	75.52 # ± 5.88	75.90 # ± 4.84	80.17 # ± 6.11	77.49 # ± 3.73	83.23 # ± 2.19	76.14 # ± 3.42	71.70 # ± 4.28	70.85 # ± 3.02	67.95 # ± 3.29

These values are expressed as the means ± SEM (*n* = 4) and analyzed by one-way ANOVA followed by Tukey’s multiple comparison test, with a simple pooled variance: Significant difference (*p* < 0.05) at different timepoints and concentrations of tannic fraction of *Stryphnodendron rotundifolium* Mart., (TFSR). Ascorbic acid (Asc. ac.). # Significant difference between the values of different concentrations at equal timepoints.

**Table 3:** TFSR Fe^3+^-reducing activity.

[TFSR] mg/mL	(%)Fe^3+^-Reducing Activity/Time (min)										Asc. ac.
	0.5	2.5	5	10	20	30	45	60	75	90	105
0.200	81.43 ±2.62	79.65 ±4.14	87.38 ±1.62	82.24 ±4.80	82.84 ±5.10	81.24 ±4.52	87.26 ±3.78	86.15 ±2.87	86.35 ±3.15	85.44 ±4.44	90.19 ± 3.99
0.100	64.70 # ± 2.09	64.18 # ± 5.40	70.34 # ± 4.84	66.10 # ± 2.53	65.01 # ± 3.32	62.91 # ± 4.19	70.31 # ± 5.17	68.58 # ± 3.39	69.80 # ± 3;06	71.66 # ± 4.49	77.69 #* ± 3.82
0.050	61.80 # ± 2.04	62.09 # ± 5.53	71.83 # ± 2.39	63.67 # ± 4.80	65.28 # ± 4.47	63.39 # ± 3.20	66.65 # ± 5.06	63.84 # ± 3.59	63.85 # ± 2.01	64.88 # ± 4.03	69.01 # ± 2.79

These values are expressed as the means ± SEM (*n* = 4) and analyzed by one-way ANOVA followed by Tukey’s multiple comparison test, with a simple pooled variance: Significant difference (*p* < 0.05) at different timepoints and concentrations of TFSR. * Significant difference between the values of equal concentration. # Significant difference between the values of different concentration at equal timepoints.

**Table 4:** 2-DR degradation.

	TFSR Protective Activity (%) ± SEM		
[2-DR] mM	0.20 mg/mL	0.10 mg/mL	0.075 mg/mL
1.50	27.22 ± 1.67	21.40 ± 1.95	17.37 ± 1.80
1.75	25.57 ± 1.87	21.09 ± 3.12	16.32 ± 1.90
2.00	24.53 ± 1.11	19.74 ± 0.49	15.46 ± 3.95
Statistical Comparison	NS	NS	NS

These values are expressed as the means ± SEM (*n* = 3) and analyzed by one-way ANOVA followed by multiple comparisons. Significant difference (*p* < 0.05) at equal concentrations of TFSR. NS No significant difference between the values.

## Data Availability

Not applicable.
